# An Uncommon Presentation of Aortic Endarteritis

**DOI:** 10.7759/cureus.52515

**Published:** 2024-01-18

**Authors:** Sofia Rito, Joao Oliveira Dias, Dina Rodrigues, Paula Martins, António Pires

**Affiliations:** 1 Pediatric Cardiology, Hospital Pediátrico do Centro Hospitalar e Universitário de Coimbra, Coimbra, PRT

**Keywords:** endocarditis, paediatric cardiology, haemorrhagic stroke, endarteritis, congenital heart disease, berry syndrome

## Abstract

Endocarditis is an uncommon infectious complication of congenital heart disease (CHD), typically presenting with fever as its primary symptom; however, its occurrence may not always be accompanied by fever. This paper elaborates on a case involving a patient with surgically corrected Berry syndrome and residual aortic coarctation. The clinical presentation of aortic endarteritis in this case manifested as seizures attributed to a hemorrhagic stroke. In this paper, we aim to raise awareness of this infrequent complication of aortic coarctation, as it may present itself with cerebral hemorrhage due to septic microemboli, even in the absence of fever at its initial presentation.

## Introduction

Patients with congenital heart disease (CHD) have an increased risk of infective endocarditis (IE) and endarteritis, particularly those with bicuspid aortic valve, coarctation of the aorta, ventricular septal defect, or tetralogy of Fallot [1]. Patients with IE are particularly prone to develop hemorrhagic and embolic events. When these events are present, a higher mortality has been reported [2].

Endocarditis typically presents with signs of systemic infection and, possibly, acute heart failure [3]. Signs of systemic infection in a patient with CHD should raise suspicions. The diagnosis of IE is based on the modified Duke criteria. Definite IE is defined as two major or one major and three minor criteria, whereas possible IE requires one major and one minor or three minor criteria [4].

Berry syndrome is a complex CHD characterized by an interrupted aortic arch, aortopulmonary window, and hemitruncus, where the right pulmonary artery arises from the aorta. Surgical correction usually involves an extensive aortic arch repair with closure of the aortopulmonary window and right pulmonary artery anastomosis to the main pulmonary artery [5]. In this report, we present a case of a child with a surgically corrected Berry syndrome, whose clinical presentation of aortic endarteritis was an afebrile hemorrhagic stroke.

## Case presentation

**Figure 1:**
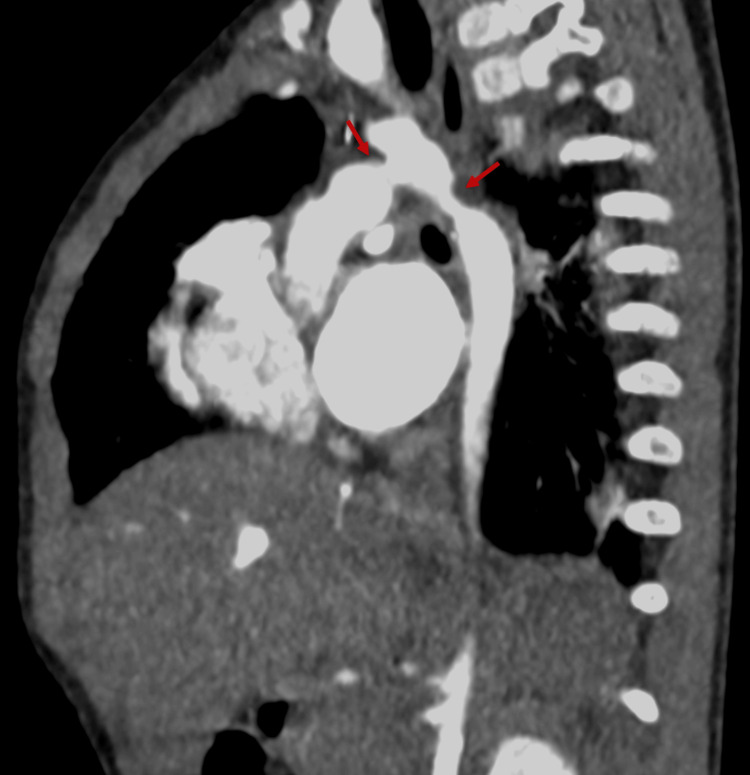
Surgically corrected Berry syndrome. Red arrows indicate the residual aortic arch coarctation after surgical correction.

**Figure 2:**
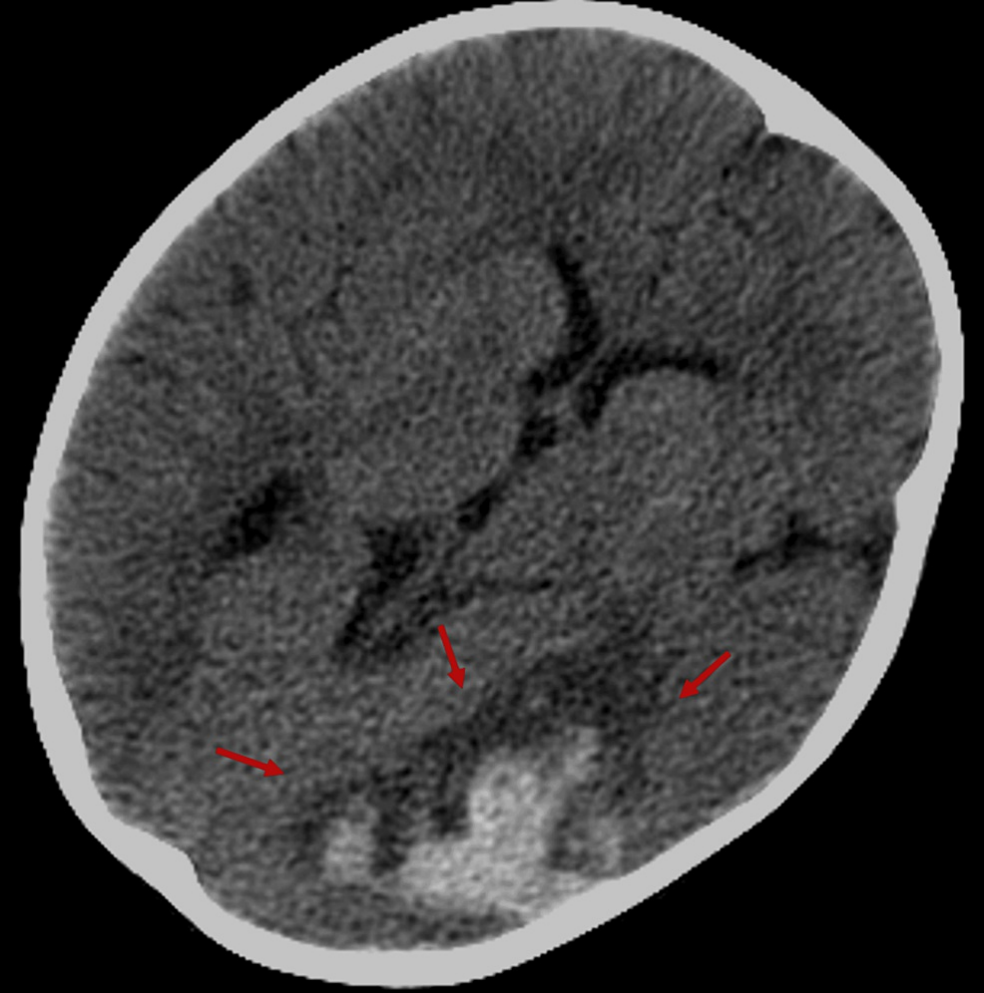
Cerebral hemorrhage. Red arrows indicate the site of a cerebral hemorrhagic stroke at admission.

A two-year-old girl, previously treated for Berry syndrome, was admitted to her local hospital after experiencing seizures characterized by bilateral tonic-clonic movements of the limbs, chewing motions, sialorrhea, and eye-rolling (Figure 1). The seizures were successfully resolved following the administration of 5 mg of rectal diazepam and 1.5 mg of intravenous midazolam. On recovery, a right hemiparesis was evident and her pupils were isocoric and reacted equally to light. The patient did not exhibit signs of respiratory distress, with SpO2 95% without supplemental oxygen. She was afebrile, with a heart rate of 120 bpm, and her blood pressure measured 101/40 mmHg. A holosystolic heart murmur graded IV/VI was evident, attributed to residual aortic coarctation. Blood tests indicated anemia with a hemoglobin level of 8.9 g/dl, a normal leukogram, and a slightly elevated protein C reactive level, while procalcitonin was negative. No relevant additional abnormalities were found. A brain CT scan was carried out, which showed a left-sided hemorrhagic stroke (Figure 2). She was started on levetiracetam 60 mg/kg and then transferred to a pediatric intensive care unit (PICU) of a tertiary hospital. On admission to the PICU, there was an improvement in her neurological state. She underwent an electroencephalogram, which showed diffuse cerebral alterations in the left hemisphere. During her stay, she became pyrexial and was empirically started on antibiotics, specifically ceftriaxone.

**Figure 3:**
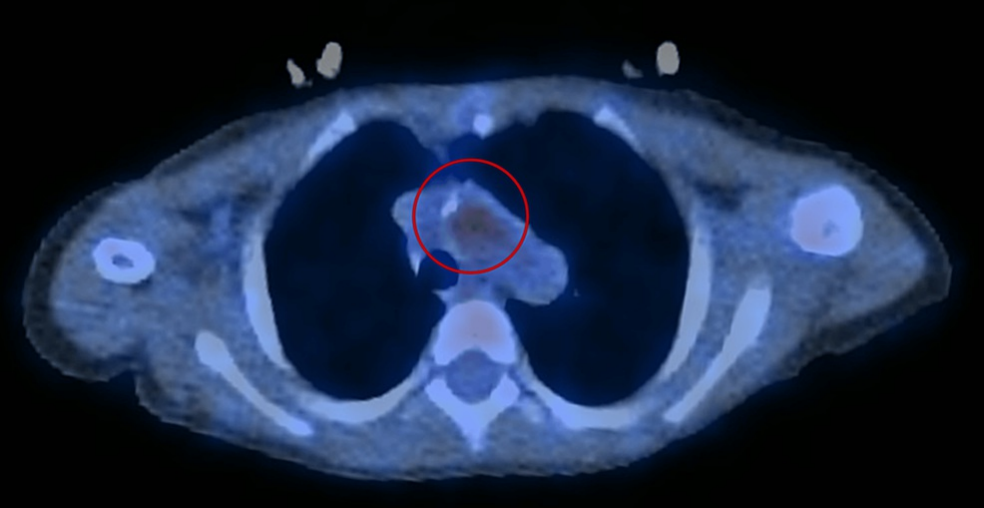
PET scan confirming the diagnosis of aortic endarteritis. PET, positron emission tomography

Although the transthoracic echocardiogram (TTE) and transesophageal echocardiogram did not show any evidence of endocarditis, all her blood cultures (BCs) tested positive for Staphylococcus epidermidis. Based on the clinical presentation and clinical signs, she met the criteria for possible endocarditis (fever, CHD, vascular phenomena, and a BC positive for S. epidermidis). Due to suspicion of aortic endarteritis, she underwent a positron emission tomography (PET) scan (Figure 3). This examination revealed discrete 18FDG uptake adjacent to and internal to the aortic wall, confirming the diagnosis of aortic endarteritis. She completed six weeks of antibiotic therapy with vancomycin and a 21-day regimen of gentamicin.

Before hospital discharge, she had negative BC and underwent a brain MRI, which showed reabsorption of the hemorrhage. Additionally, evidence of cortical and subcortical microhemorrhages was observed, likely associated with embolic events. Due to significant clinical improvement and the absence of seizure events, the decision was made to discontinue anticonvulsant therapy.

## Discussion

Aortic coarctation creates a favorable anatomical condition for the development of endarteritis due to the change in the blood flow dynamics. This change in flow may result in endothelial damage, increasing the susceptibility to endovascular infections [6,7], possibly triggered by shear stress forces. Aortic grafts also play an important role in the development of endarteritis. In patients with ascending aortic graft infections, multimodality imaging has an important role in both diagnosis and follow-up [8].

Although fever is the main symptom of endarteritis and/or endocarditis, it may be absent in the initial stages of the disease. Clinicians require a high index of suspicion to direct diagnostic procedures toward endarteritis in the setting of a hemorrhagic stroke in pediatric patients [7,9].

Patients with infective endocarditis can have multiple neurological complications, including cerebrovascular events, transient ischemic attack, meningitis, brain abscess, seizure, and mycotic aneurysm. Intracranial hemorrhage accounts for 5% of neurological complications in IE [9].

Our patient presented with a cerebral vascular event, despite the absence of the most common risk factors associated with embolic infarcts. These include older age, the presence of atrial fibrillation, multiple valvular endocarditis, mitral valve vegetation, and larger vegetation [2]. Neurovascular events, particularly due to a septic embolus, should probably have a more important role in the diagnosis of IE, as it affects the patient’s prognosis.

## Conclusions

In conclusion, aortic endarteritis is a rare entity, even in the presence of CHD. In the absence of signs suggestive of endocarditis on TTE, other complementary diagnostic examinations, such as a PET scan, may be necessary to confirm the diagnosis.

We aim to raise awareness of this uncommon complication of aortic endarteritis, as it may manifest with seizures resulting from septic microemboli, even in the absence of fever at the initial presentation. The authors believe that this is the first published case of aortic endarteritis complicated by a cerebrovascular accident in the pediatric population.
